# Prevalence of Cardiovascular Risk Factors in Spain: A Systematic Review

**DOI:** 10.3390/jcm12216944

**Published:** 2023-11-06

**Authors:** Jennifer Sacramento-Pacheco, María Begoña Sánchez-Gómez, Juan Gómez-Salgado, María Mercedes Novo-Muñoz, Gonzalo Duarte-Clíments

**Affiliations:** 1 Nuestra Señora de Candelaria Nursing University School, University of La Laguna, 38010 San Cristóbal de La Laguna, Spain; 2 Europa Sur Educational Centre (CESUR Tenerife), 38006 Santa Cruz de Tenerife, Spain; 3 Cátedra de Enfermería, Universidad de La Laguna, 38200 San Cristóbal de La Laguna, Spain; 4 Cieza Este Health Centre, Area IX, Servicio Murciano de Salud, 30530 Murcia, Spain; 5 Department of Sociology, Social Work and Public Health, Faculty of Labour Sciences, University of Huelva, 21071 Huelva, Spain; 6 Safety and Health Postgraduate Program, Universidad Espíritu Santo, Guayaquil 092301, Ecuador; 7 Department of Nursing, Faculty of Health Sciences, University of La Laguna, 38200 San Cristóbal de La Laguna, Spain; 8 Case Management, Area IX, Servicio Murciano de Salud, 30530 Murcia, Spain

**Keywords:** heart disease, cardiovascular disease, prevalence, cardiovascular risk factors, Spain

## Abstract

Cardiovascular diseases are the leading cause of death in Spain, according to data from the National Institute of Statistics, with the lack of control of cardiovascular risk factors (CVRF) being the main contributing factor. The CVRFs of greatest clinical interest are high blood pressure (HBP), smoking, diabetes mellitus (DM2), overweight, obesity, hypercholesterolaemia, and sedentary lifestyle. The main objective of this review was to compare the prevalence of the different CVRFs according to population-based studies carried out in Spain. For this, a systematic review based on publications assessing CVRFs in the adult population and estimating their national prevalence was conducted. Pubmed and Dialnet databases were consulted, and the selected articles were analysed using the Critical Appraisal Skills Programme Español (CASPe) tool for cohort studies and the Berra et al. tool for cross-sectional studies. A total of 33 studies were obtained from the autonomous regions of Andalusia, the Canary Islands, Castilla-Leon, Castilla-La Mancha, Catalonia, Extremadura, the Balearic Islands, Madrid, Murcia, and Navarra. In all the population-based studies, there was a greater representation of women in the sample. The most prevalent CVRFs differed across the studies according to the autonomous region targeted, with dyslipidaemia, sedentary lifestyle, high blood pressure, hypercholesterolaemia, overweight, and obesity standing out. Numerous differences exist between the studies included in this review, such as the age range, the CVRFs analysed and their prevalence, and remarkable aspects such as the over-representation of the female sex in all cases. It can be concluded that, based on the presented results, the prevalence of CVRFs in Spain varies according to the autonomous region, the sex of the individual, and the studied age range.

## 1. Introduction

### 1.1. Theoretical Basis

Cardiovascular diseases (CVDs) are the leading cause of death in Spain, according to data from the National Institute of Statistics [1]. If the mortality rate is disaggregated by sex, it can be seen that diseases associated with the circulatory system have been the leading cause of death in women and the second leading cause in men in recent decades [2,3,4].

Looking at deaths due to CVD by age group and sex, acute myocardial infarction (AMI) stands out as a pathology with high mortality rates and which is more prevalent in men, although a decrease in the mortality associated with this cause has been observed in recent years. The latest data on heart failure, meanwhile, place it above deaths due to AMI, finding higher mortality rates in women in this case. In addition, an increasing trend in deaths associated with heart failure can be observed. As regards cerebrovascular pathologies, mortality rates are high in both sexes, being greater in women, than in the case of deaths due to AMI, in which a decreasing trend is observed. A causal relationship between mortality and age is also identified [5].

CVDs could be prevented by adequately controlling cardiovascular risk factors (CVRFs). In Spain, they are highly prevalent and, in many cases, inadequately controlled. It is estimated that at least 80% of cardiovascular diseases could be preventable [3,4]. Prevalence can be defined in this context as the number of cases of a disease in a specific population at a particular timepoint or over a specified period of time [5].

A CVRF can be defined as a biological characteristic or a habit or lifestyle that increases the likelihood of developing a CVD or of dying from a CVD in those individuals who exhibit it [6]. The main CVRFs are classified as modifiable or non-modifiable. Non-modifiable risk factors are those inherent to the individual, and these include sex, age, genetic or family history, and race. In contrast, modifiable risk factors are those that can be controlled, and these are the ones that are of greatest clinical interest and are assessed in different population studies, notably high blood pressure (HBP), smoking, type 2 diabetes mellitus (DM2), overweight, obesity, hypercholesterolaemia, and sedentary lifestyle [7,8].

The risk of developing a CVD can be estimated using tools that allow quantification of the actual probability of developing a CVD and that contribute to guide the most suitable treatment and monitoring for each patient on an individual basis [8].

Nurses are actively involved in health-promotion and CVD-prevention interventions aimed at encouraging healthy lifestyle habits [8]. Therefore, one of the main nursing competencies must be to be able to use appropriate prevention strategies aimed at cardiovascular risk factors (CVRFs) to reduce the incidence and prevalence of both CVDs and CVRFs. This is done by first identifying the occurrence of CVRFs and, then, controlling for them and adjusting them to the level of care required for each patient. This provision of care is encompassed in the nursing diagnosis ‘Risk of impaired cardiovascular function’ of the NANDA-I diagnostic taxonomy.

Given the variability found across research at the national level regarding the studied CVRFs, a comparison of the different studies is necessary in order to obtain their prevalence in the different communities. In this way, it should be possible to establish a comparison across the Spanish adult population and thus understand the current situation and the changes that have occurred over the years. Epidemiological data are also needed to estimate the population targeted through such interventions. Although studies exist in different national and international contexts in this regard, at the national level, it is important to be able to rely on data adapted to each population group.

### 1.2. Objective

To compare the prevalence of CVRFs according to population-based studies carried out in Spain.

## 2. Materials and Methods

### 2.1. Protocol

A systematic review was conducted following the Preferred Reporting Items for Systematic Reviews and Meta-Analyses (PRISMA) [9], which sets out the quality requirements to be met in a review.

The systematic review process consisted of several stages: establishing the research question by applying the PICO format (P: patient; I: intervention; C: comparator; O: outcome); developing the protocol for inclusion and exclusion criteria for citations; conducting the literature search; selecting the studies [10] through the Critical Appraisal Skills Programme Español (CASPe) [11] critical reading tool, the Berra et al. [12] tool, and synthesis of the evidence [13]. This way, the best available evidence was identified, assessed, and synthesised, and possible information gaps were defined in order to resolve future unanswered questions. The PICO-formatted research question can be found in Table 1.

The protocol has been registered in Open Science Framework (OSF) and can be accessed via https://doi.org/10.17605/OSF.IO/JHMSZ (accessed on 16 October 2023) [14].

### 2.2. Eligibility Criteria

- Inclusion criteria

All publications assessing cardiovascular risk factors in the adult population over 18 years of age and estimating their prevalence in different Spanish autonomous regions were included.

- Exclusion criteria

Publications whose methodology was not explicit, those that did not obtain conclusive results, did not estimate prevalence of cardiovascular risk factors, and those whose results were not applicable in different study contexts were excluded.

Publications in English and Spanish were used as search limits.

### 2.3. Sources of Information

The electronic databases consulted were Pubmed and the Virtual Health Library (VHL). The search was completed with a reference search. The last search date was 8 August 2023.

### 2.4. Search Strategy

The following search equations were used: ‘Cardiovascular Risk Factors AND Prevalence AND Spain’ and ‘Heart Disease Risk Factors AND Prevalence AND Spain’. The search strategy is detailed in Table 2.

### 2.5. Selection of Studies

Following the PRISMA guidelines [15,16], screening was carried out by reading titles and abstracts. Full articles were critically read and assessed using the CASPe tool for cohort studies [11], the Berra et al. tool for cross-sectional studies [12], and also synthesis of the evidence [13]. This analysis is detailed in Table 3 [2,3,17,18,19,20,21,22,23,24,25,26,27,28,29,30,31,32,33,34,35,36,37,38,39,40,41,42,43,44,45,46,47].

### 2.6. Data Collection

Ad hoc forms were created, which allowed researchers to independently review the main characteristics of each included study and peer-review all studies. All data considered relevant for the writing and synthesis of the different sections of the study were extracted according to the objectives and compared in order to resolve any possible discrepancies between the different reviewers.

### 2.7. Data Aggregation

The results obtained were grouped based on the CVRFs exposed in each study by autonomous region and are presented as a percentage of the total population included in each study, differentiated by sex (in cases where these data were present) and indicating the total prevalence. In cases where the total prevalence of CVRFs in the sample was not available, but the prevalence by sex and the percentage of participants of each sex were known, the prevalence was calculated using a mathematical equation.

### 2.8. Risk of Study Bias

The assessment of the risk of bias was conducted after critical screening with the CASPe tool for cohort studies [11], obtaining a minimum score of 9 out of 10. In the case of cross-sectional studies, the Berra et al. tool was used [12], obtaining high quality in all cases. The grouped results of this critical appraisal are shown in Table 3.

### 2.9. Synthesis Methods

The synthesis of the evidence was conducted according to the Scottish Intercollegiate Guidelines Network (SIGN) [13], obtaining 2++ levels of evidence (LE) and B degrees of recommendation (DR) according to the LE. The results of this synthesis are shown in Table 3.

### 2.10. Reporting the Risk of Bias

The evidence was critically read and synthesised to ensure that the included studies were free of possible biases; the synthesis of the evidence was carried out by a third-party reviewer.

### 2.11. Certainty Assessment

The certainty of the critical reading and the synthesis of the evidence were supported by the critical reading tool (CASPe) [11], the Berra et al. [12] tool, and the synthesis of the evidence based on the SIGN [13]. The critical reading and the synthesis of the evidence were carried out by means of ad hoc, double-blind questionnaires. This guaranteed the certainty of the degrees of recommendation established on the basis of the available evidence.

## 3. Results

### 3.1. Selection of Studies

**Figure 1:**
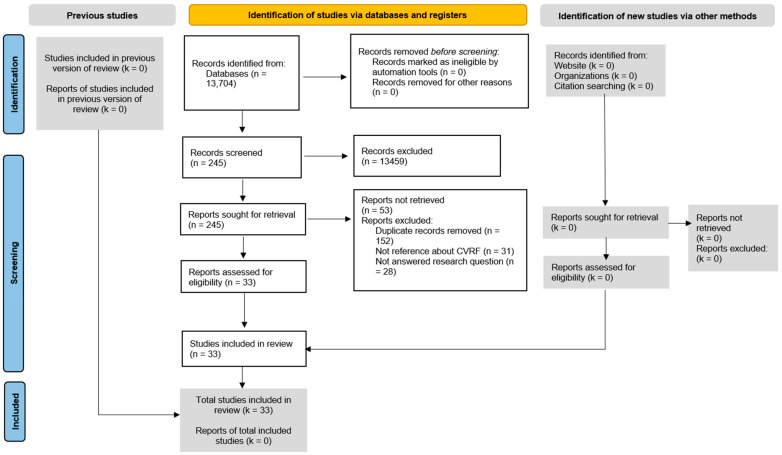
PRISMA flow diagram.

Figure 1 shows the selection process of the references used [15,16]. The selected bibliographic references were mainly descriptive cross-sectional studies and cohort studies [2,3,17,18,19,20,21,22,23,24,25,26,27,28,29,30,31,32,33,34,35,36,37,38,39,40,41,42,43,44,45,46,47].

**Figure 2:**
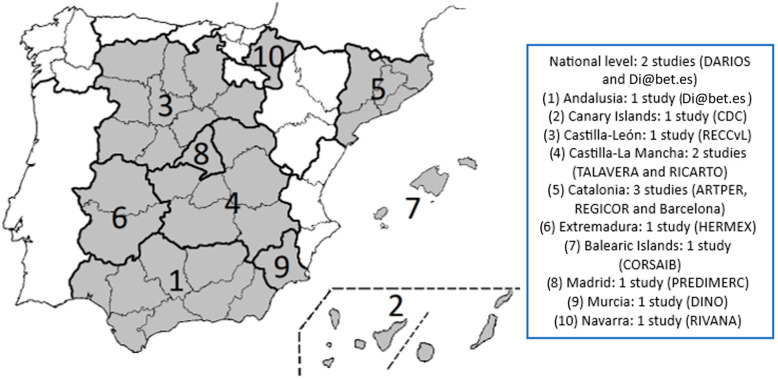
Spanish autonomous regions participating in the study. Source: self-elaborated.

The studies were conducted between 1999 and 2020. The results show the different findings taken from these Spanish population-based studies. Results were obtained from studies carried out in Andalusia, the Canary Islands, Castilla-Leon, Castilla-La Mancha (Toledo and Talavera), Catalonia (Barcelona and Girona), Extremadura, the Balearic Islands, Madrid, Murcia, and Navarra, and at the national level. Figure 2 shows the Spanish autonomous regions participating in the study.

### 3.2. Individual Results of the Studies by Autonomous Region and Characteristics

Dyslipidaemia, Atherosclerosis Risk and increased hsCRP and Inflammatory and Oxidative status in the Spanish population (DARIOS, comparison at national level) [17,18,19,20].

The DARIOS study was a pooled analysis with individual data from 11 population-based studies, conducted from 2000 onwards, which included 27,903 participants aged 35–74 from 10 Spanish autonomous regions. Of the sample, 54% percent were women and 46% were men. The participation in the study was quite balanced in the 35–44 (27.3%), 45–54 (26.5%), and 55–64 (27.1%) age groups, with a decreasing prevalence in the 65–74 age group (19.1%) [17].

Regarding cardiovascular risk, 11% of the men included in the study were at high or very high risk, and 2.3% of the women were at high or very high risk. CVR was measured with the calibrated REGICOR function [17].

Regarding the prevalence of CVRFs, 33.5% of the participants had dyslipidaemia, 29.3% had high blood pressure, and 13.6% had DM2 [17].

With regard to high blood pressure, DM2, and smoking, it was observed that the higher the prevalence of these factors, the higher the cardiovascular risk [17].

In the group of hypertensive men, it can be observed that those with low cardiovascular risk had the highest pharmacological control of the disease, which translated into 32% of hypertensive men being treated and controlled. In contrast, diabetic men with a high cardiovascular risk were those with the highest percentage of disease control, at 40%. Women, both hypertensive and diabetic, who had greater control of the disease, had a low cardiovascular risk [17].

In all cases, those participants previously diagnosed with any of the CVRFs were included as patients with that CVRF. Those taking antihypertensive drugs or under dietary control, or those with blood pressure levels above 140/90 mmHg were considered hypertensive. The percentage of diabetic participants corresponded to patients on treatment or with fasting blood glucose levels above 126 mg/dL. Hypercholesterolaemia was present in patients receiving pharmacological treatment or dietary control for hypercholesterolaemia, taking into account that participants were considered controlled if, on the one hand, they had a LDLc < 100 mg/dL in patients with a high cardiovascular risk, or were diabetic, or had a LDLc < 115 mg/dL in the rest of patients; on the other hand, they were considered controlled if the patient was diabetic, <130 mg/dL if the patient had a high or moderate cardiovascular risk, and <160 mg/dL if the patient had a high cardiovascular risk [17].

In the 2014 study by Baena-Díez et al., which aimed to determine the prevalence of atrial fibrillation and other associated CVRFs in Spain, a cross-sectional study was carried out in which 17,291 individuals belonging to six population-based studies were included [18].

Of the 17,291 individuals included in the study, 47.1% were men and 52.9% were women. The mean age was 57 years, with a SD of 13. This study did not disaggregate prevalence by sex and it was found that 35.2% of participants had high blood pressure, 13.2% had DM2, 37% had hypercholesterolaemia, and 22.9% were smokers. In addition, 43% were overweight and 29.6% were obese [18].

The prevalence of ischaemic heart disease was 2.8%, of heart attack it was 2.4%, and of atrial fibrillation it was 1.5% [18].

Regarding the prevalence of CVRFs in the 2014 cohort, the most prevalent CVRF was overweight, with a prevalence of 43%, followed by hypercholesterolaemia (37%), high blood pressure (35.2%), obesity (29.6%), smoking (22.9%), and DM2 (13.2%) [18].

Hypertensive patients were considered those previously diagnosed, taking antihypertensive drugs or under dietary control, or those with blood pressure levels above 140/90 mmHg. The percentage of diabetic participants corresponded to patients diagnosed, under treatment, or with fasting blood glucose levels above 7 mmol/L. Hypercholesterolaemia was considered present in those participants who had been previously diagnosed or had cTotal > 6.2 mmol/L. In the case of hypertriglyceridaemia, the included subjects had triglyceride values greater than 1.7 mmol/L. The diagnosis of overweight and obesity was based on the Body Mass Index classification (BMI) [18].

Another study using the population-based cohort of the DARIOS study of 24,670 individuals showed that there was a higher prevalence of metabolic syndrome (MS) in men than in women, where out of 7832 participants suffering from MS, 32% were men and 29% were women. By autonomous region, according to this study, the highest prevalence of MS in men corresponded to the Balearic Islands, the Canary Islands, and Extremadura, and in women, to the Canary Islands, the Balearic Islands, and Castilla-Leon. Participants with a higher prevalence of MS were also found to have a higher prevalence of CVD and DM2, which applied to both sexes, with women having a higher prevalence of smoking and lower educational level [19].

On the other hand, the 10-year cardiovascular risk was higher in men, both in those with and without MS.

By age group, the prevalence of MS was highest in men up to the age of 54 years, was equal in the 55–64 age group and, from the age of 65 years onwards, it was women who had the highest prevalence. The reported prevalence of MS was 19.7% for men aged 35–44 years and 10.9% for women that age; 31.7% for men and 24.9% for women aged 45–54 years; 40.6% for men and 42.1% for women aged 55–64 years; and 42.2% for men and 52.5% for women aged 65–74 years [19].

In this case, tobacco use was measured according to reported smoking. Overweight and obesity were measured according to the BMI classification and waist circumference measurement. Blood pressure figures were determined, and triglyceride, HDL-C, and glucose values were also assessed. For participants to be diagnosed with MS, they had to meet three of the five established criteria: fasting blood glucose > 100 mg/dL; or treatment with insulin or hypoglycaemic agents; treatment with antihypertensive drugs or blood pressure above 130/85 mmHg; HDL-C < 50 mg/dL in women and <40 mg/dL in men; triglycerides > 150 mg/dL; and abdominal circumference > 88 cm in women and >102 cm in men [19].

A study published in 2013 showed the prevalence of overweight and obesity by sex in a total of 28,887 participants. The prevalence of overweight in men was 50.7% and 35.6% in women. For obesity, it was slightly higher in women, with a prevalence of 28.3%, as compared to men, with a prevalence of 28%. The overall prevalence of overweight in this study was 42.6% and 28.2% for obesity [20].

All participants were measured for waist circumference, weight, and height. The prevalence of overweight and obesity was estimated according to the BMI classification [20].

Di@bet.es study (Andalusia) [21,22,23].

The Di@bet.es study is a cross-sectional population-based study that assessed the prevalence of cardiovascular risk factors and their association with lifestyle in Andalusia, and compared it with the rest of Spain [21].

The final study sample in Andalusia was 1517 individuals, of whom 64.9% were women and 35.1% men. The final study sample in the rest of Spain was 3586 individuals, where 53.8% were women and 46.2% were men [21].

In terms of the prevalence of CVRFs, all had a higher prevalence in Andalusia compared to the prevalence at national level. The most prevalent CVRF in the Andalusian population sample was low level of physical activity (58.9%), followed by hypercholesterolaemia (50.3%), high blood pressure (43.9%), obesity (37%), smoking (29%), and DM2 (16.3%). In the rest of Spain, the most prevalent risk factor was hypercholesterolaemia (49.3%), followed by high blood pressure (39.9%), low level of physical activity (35.8%), smoking (27.4%), obesity (26.6%), and diabetes (12.5%) [21].

Based on the population-based cohort of the Di@bet.es study, the prevalence of CVRFs was estimated at national level. A study assessing the incidence of DM2 and which showed the prevalence of different CVRFs was published in 2020. It concluded that, out of the initial sample of 5072 study participants, only 2408 of them were re-evaluated. From these results, it was found that 39.7% of those re-evaluated were men and 60.3% were women. Of the CVRFs presented, the one with the highest prevalence was sedentary lifestyle or low level of physical activity, with an overall prevalence of 42.3%, followed by overweight (42.2%), high blood pressure (38.8%), dyslipidaemia (26.6%), obesity (26.2%), smoking (24.4%), and DM2 (6.5%) [21,22].

A participant who reported smoking one or more cigarettes per day was considered to be a smoker. In the case of DM, prevalence was calculated as known DM plus unknown DM, taking into account that the oral glucose overload test was performed on those participants who consented to it, with those who did not consent to it being adjusted by statistical formula. High blood pressure was diagnosed in participants on antihypertensive treatment or with blood pressure levels >140/90 mmHg. Overweight and obesity were determined according to the BMI classification and, for obesity, abdominal circumference was also determined. Hypercholesterolaemia was defined as a total concentration greater than 200 mg/dL. Dyslipidaemia was defined as triglyceride levels greater than or equal to 1.7 mmol/L or HDL-C below 1.29 mmol/L in women and 1.03 mmol/L in men, or if medication was being used [21,22].

Also in the same year, a study was published in which the re-evaluated population (2408) was taken as a reference; a sample of 1881 participants was extracted from this population. These 1881 participants were divided into two groups: group A, where there was an absence of MS; and group B, where MS was identified, represented by 39% of the participants. Of the total sample, 42.6% were male and 57.4% were female. Of the total male participants, 52.1% had MS; of the total female participants, 47.9% had MS [23].

Metabolic syndrome was defined using the waist circumference measurement as defined for the Spanish population, set above 94.5 cm in men and 89.5 cm in women as being pathological; with blood pressure levels >130/85 mmHg; triglycerides >150 mg/dL; HDL-C <50 mg/dL in women and <40 in men; and glucose >100 mg/dL. Subjects who were under treatment for some of the CVRFs were also included as subjects with CVRFs [23].

CDC CANARIAS (CARDIOVASCULAR, DIABETES, CÁNCER) (Cardiovascular diseases, Diabetes, and Cancer study in the Canary Islands) [24,25,26,27].

This cohort study on the general population analysed the prevalence and incidence of cardiovascular diseases and exposure to the associated risk factors in the adult population of the Canary Islands [24].

The cohort study was published in 2008. The population cohort was assigned by random sampling in which 6718 participants between 18 and 75 years of age participated, with the highest percentage of participation corresponding to the island of Tenerife, followed by Gran Canaria, El Hierro, Lanzarote, La Palma, La Gomera, and Fuerteventura [24].

Of the total number of participants, 43% were men and 57% were women. In the case of Tenerife, 48% (1234) were men and 52% (1339) were women; in Gran Canaria, 37% men (822) and 63% (1423) women participated; El Hierro had 49% (225) of men and 51% (239) of women; Lanzarote had 40% (158) of male participants and 60% (239) of female participants; in La Palma, 43% (168) were men and 57% (227) were women; in La Gomera and Fuerteventura, 46% were men (158 and 134, respectively) and 54% were women (184 and 159, respectively) [24].

In terms of risk factors by age, there were differences between men and women. Prevalence data for DM2, high blood pressure, hypercholesterolaemia, MS according to the International Diabetes Federation (IDF), overweight, smoking, and alcohol abuse were higher in men. Prevalence data for MS according to the NCEP Adult Treatment Panel III (ATP III), obesity, and sedentary lifestyle were higher in women [24].

In men, DM2 had an overall prevalence of 12%; high blood pressure, 43%; hypercholesterolaemia, 32%; ATP III metabolic syndrome, 24%; IDF metabolic syndrome, 37%; obesity, 27%; overweight, 45%; sedentary lifestyle, 55%; smoking, 31%; and alcohol abuse, 13%. In the case of DM2, hypertension, hypercholesterolaemia, MS, and sedentary lifestyle, an increasing trend with age was observed. In women, DM2 had an overall prevalence of 10%; of 33% for high blood pressure; for hypercholesterolaemia, 31%; ATP III metabolic syndrome, 24%; IDF metabolic syndrome, 31%; obesity, 29%; overweight, 33%; sedentary lifestyle, 71%; smoking, 21%; and alcohol abuse, 2%. In the case of DM2, high blood pressure, hypercholesterolaemia, MS, overweight, and sedentary lifestyle, there was an increasing trend with age. In both sexes, obesity was more prevalent in the 45–65 age group and smoking in the 30–45 age group [25].

The most prevalent CVRF in men was sedentary lifestyle, followed by overweight, high blood pressure, IDF metabolic syndrome, hypercholesterolaemia, smoking, obesity, ATP III metabolic syndrome, alcohol abuse, and DM2. In the case of women, sedentary lifestyle was also the most prevalent CVRF, followed by high blood pressure and overweight, IDF metabolic syndrome and hypercholesterolaemia, obesity, ATP III metabolic syndrome, smoking, DM2, and alcohol abuse [24,25].

With regard to the total prevalence of CVRFs, the most prevalent factor was sedentary lifestyle, with a total prevalence of 64%, followed by overweight (38.2%), high blood pressure (37.3%), hypercholesterolaemia (31.4%), obesity (28.1%), smoking (25.3%), MS (24%), DM2 (10.6%), and alcohol abuse (6.7%) [24,25].

Subsequently, in 2020, Cuevas Fernández et al. published an article based on the CDC study cohort, following up the cohort to assess the prevalence of smoking. The total population had decreased, with a population of 6729 in 2000, 6171 in 2008, and 4705 in 2015. This led to a change in the percentages by sex, with 59% female participants compared to 41% male participants in 2015. Smoking prevalence had decreased in both sexes, from a prevalence of 31% in men and 21% in women in 2000 to a prevalence of 25% in men and 18% in women in 2015, although the prevalence remained higher in men than in women [26].

A subsequent study published in 2016 aimed to obtain a valid, simple, and low-cost scale to measure tobacco exposure only in terms of duration, with which to promote readiness to change in order to initiate timely preventive intervention. The Canary Islands CDC study cohort was taken as a reference, of which 6172 participants were recruited for this subsequent study, 57% of whom were women and 43% were men. Of the total number of participants, 24% were active smokers, 62.3% had lived with smokers at home, and 35.3% had shared a workplace with smokers [27].

Any participant with a previous diagnosis and/or being treated for diabetes, or with a fasting blood glucose >125 mg/dL after a second confirmatory test was considered diabetic; to determine blood pressure, figures greater than or equal to 140/90 mmHg were taken as reference, and those participants previously diagnosed were also included in this group. All subjects who had been previously diagnosed with hypercholesterolemia or with total cholesterol levels greater than 240 mg/dL were considered to have hypercholesterolemia (HDL-C values below 40 mg/dL in men and 50 mg/dL in women were examined). For MS, the ATPII and IDF definitions were used; tobacco and alcohol consumption were determined when participants declared to do so, and sedentary lifestyle when participants reported daily physical activity of less than 30 min. For obesity and overweight, the BMI classification was used [24,25,26,27].

Cardiovascular Disease Risk Study in Castilla-Leon (RECCyL, Castilla-Leon) [28,29,30].

The RECCyL study was a population-based cross-sectional study involving Primary Care professionals in Castilla-Leon.

The final study sample consisted of 4012 participants from rural, semi-urban, and urban areas of the Castilla-Leon autonomous region. In total, 48.1% were men and 51.9% were women. Of the total sample, 45.6% were from a rural area and 54.4% from an urban or semi-urban area. The age group with the highest prevalence of participation was 35–64 years, with a prevalence of 45.9%, followed by the age group over 65 years with a prevalence of 31.9%, and finally the 15–34-year age group, with a prevalence of participation of 22.2% [28].

A study published in 2008, which assessed the prevalence of high blood pressure, took as a reference the sample of 4012 participants of the RECCyL study. This study showed the prevalence of total high blood pressure by sex and age group and by sex and area, and also related high blood pressure to other CVRFs [28].

The prevalence of high blood pressure was 40.4% in men and 37.4% in women, with an overall prevalence of 38.7% in the total sample. As can be seen, the prevalence was higher in men than in women. When disaggregated by geographical area, prevalence in both sexes was higher in rural areas, and there was still a higher prevalence in men [29].

Regarding the prevalence of high blood pressure by age group and sex, in the case of men, an increasing trend was observed in all age groups except in the 55–59 age group. The age group with the highest prevalence was 75 years and older; in women, there was also an increasing trend except in the 65–69 age group. Comparing both sexes, the prevalence of high blood pressure in men was higher than in women from 15 to 54 years of age, this difference being particularly notable in the 35–44 age group. From the age of 55 onwards, high blood pressure was more prevalent in the female sex, except in the 70–74 age group, where the prevalence was almost the same in both sexes [29].

As for the other CVRFs, when related to high blood pressure, there was a higher prevalence of these in patients with high blood pressure, with the exception of smoking, which was higher in patients without high blood pressure. The most prevalent pathology in hypertensive participants was hyperlipidaemia (44%), followed by obesity (36.7%), MS (31.7%), DM2 (18.4%), and smoking (13.7%). In non-hypertensive patients, the prevalence of CVRFs was the same, with the exception of smoking, which was at the top of the list [29].

Regarding the prevalence of CVRFs, the most prevalent was high blood pressure (38.7%), followed by hyperlipidaemia (34%), obesity (27.1%), smoking (20.6%), MS (20.5%), and DM2 (12.3%).

In 2010, a study was published based on the population-based cohort of the RECCyL study, in which the prevalence of obesity was studied. The total prevalence of obesity was 21.7%, being higher in women (23.2%) than in men (20.4%). Regarding morbid obesity, the total prevalence was 1.4%, being higher in women (1.9) than in men (1%). The total prevalence of obesity was higher in rural areas (26.2%) than in urban or semi-urban areas (18.7%) [30].

Regarding obesity by age group and sex, in the 15–49 and 55–59 age groups, it was more prevalent in the male sex, but from the age of 50 onwards, the prevalence increased notably in women. Regarding the total prevalence of obesity, the 60–64 age group had the highest prevalence of obesity [30].

As regards morbid obesity, the prevalence was lower than for obesity, but with the difference that it was more prevalent in women in all age groups except in the 45–49 and 60–64 age groups, where the prevalence was higher in men. In the 50–54 age group, the prevalence in women was higher than in the other age groups [30].

Obesity was compared with the prevalence of other CVRFs and a higher prevalence of CVRFs was observed in obese patients compared to those who were overweight or at their normal weight. The only CVRF that decreased was smoking [30].

Participants who had a previous diagnosis of high blood pressure, were under treatment, or had blood pressure levels above 140/90 mgHg were considered to have high blood pressure; those with a previous diagnosis or with fasting glucose levels >125 mg/dL were considered diabetics; hyperlipidaemia was considered when total cholesterol was greater than 250 mg/dL; smokers were defined as those who reported smoking one or more cigarettes per day; obesity was diagnosed according to the BMI classification; MS was defined according to ATPIII criteria; waist circumference was defined as pathological as greater than or equal to 102 cm in men and 88 cm in women [29,30].

TALAVERA study (Castilla-La Mancha).

The TALAVERA study was carried out in Talavera de la Reina, Toledo, Castilla-La Mancha. The study included a total of 1330 individuals, of which 47.3% were men and 52.7% women. The participants were aged between 25 and 74 years and the age group with the highest participation was 55–64 years for women and 65–74 years for men [31].

Regarding the prevalence of CVRFs, the prevalence of hypercholesterolaemia (16.8% in women and 14.4% in men), high blood pressure (42.1% in women and 40.8% in men), and obesity (37.8% in women and 21.2% in men) was higher in women than in men, while the prevalence of smoking (39.4% in men and 13.7% in women) was higher in men than in women. The most prevalent CVRF was high blood pressure, with a total prevalence of 41.5%, followed by obesity (29.9%), smoking (25.9%), and hypercholesterolaemia (15.7%) [31].

Obesity was defined according to the BMI classification. All subjects with a previous diagnosis of high blood pressure or under treatment, or whose blood pressure exceeded 140/90 mgHg were considered hypertensive. Hypercholesterolaemia was defined as total cholesterol levels greater than or equal to 250 mg/dL and HDL-C below 35 mg/dL [31]. 

RIesgo CARdiovascular y eventos cardiovasculares en la población general del área sanitaria de TOledo (cardiovascular risk and cardiovascular events in the general population of the Toledo health area) study (RICARTO, Toledo).

The RICARTO study is an observational epidemiological study in which the population over 18 years of age of Toledo was included. The aim of the study was to determine the prevalence of CVRFs, subclinical organ damage, and CVD, as well as the degree of control for CVRFs in a randomised sample [3].

The calculated sample size was 3125 cases. The results of the first 1500 were presented. Of these, 55.6% were female and 44.4% were male. Participants over 18 years of age were included, stratified by age groups: 41.8% of participants between 18 and 44 years; 39.9% between 45 and 64 years; 15% between 65 and 79 years; and 3.3% over 80 years of age. Regarding the female sex, there was a higher prevalence of participants in the 45–64 years age range, and as for males, in the 18–44 years age range. In both men and women, the highest percentage of the population studied came from rural areas, 64% of women and 64.1% of men [3].

Regarding the prevalence of CVRF by sex, in both men and women, the most prevalent CVRF was dyslipidaemia, being more prevalent in men (60.9%) than in women (53.7%). In second place for men was overweight (45.1%), followed by high blood pressure (40.4%), sedentary lifestyle (33.8%), obesity (27.7%), smoking (25.8%), MS (23.7%), diabetes (11, 2%), and alcohol abuse (4.7%). For women, sedentary lifestyle (43.8%), overweight (34.3%), high blood pressure (27.1%), obesity (23.4%), smoking (22.9%), MS (17.2%), diabetes (6.4%), and alcohol abuse (0.4%) were more prevalent. It can be seen that there was a higher prevalence of CVRFs in men except for sedentary lifestyle, which was higher in women. Regarding the total prevalence of CVRFs, the most prevalent was dyslipidaemia, with a total prevalence of 56.9%, followed by sedentary lifestyle (39.4%), overweight (39.1%), high blood pressure (33%), obesity (25.3%), smoking (24.2%), MS (20.1%), diabetes (8.6%), and alcohol abuse (2.3%) [3].

Hypertension was determined for individuals with a diagnosis, or under treatment, or with blood pressure greater than or equal to 140/90 mgHg. DM was defined as being diagnosed or under treatment, or showing fasting blood glucose levels greater than 126 mg/dL or glycosylated haemoglobin ≥ 6.5%. The diagnosis of obesity was based on the BMI classification and abdominal obesity when the waist circumference exceeded 88 cm in women and 102 cm in men. Dyslipidaemic individuals were those with a previous diagnosis, under lipid-lowering treatment, or with total cholesterol ≥ 200 mg/dL, or triglycerides ≥ 200 mg/dL, or both. Participants were considered smokers if they had consumed at least one cigarette in the last month. Alcohol abuse and sedentary lifestyle were established as reported by the participant. To determine MS, at least three of the following criteria had to be met: fasting blood glucose ≥ 100 mg/dL or being treated with oral anti-diabetic drugs or insulin; blood pressure ≥ 130/85 mmHg or being treated; HDLc <50 mg/dL in women and <40 in men; or waist circumference ≥ 102 in men or ≥ 88 cm in women [4].

Peripheral Arterial Disease study (PERART/ARTPER, Barcelona).

The ARTPER study was a prospective study with 3786 participants over the age of 49, of whom 46.1% were males and 53.9% were females [32]. The prevalence of CVRFs was presented excluding patients with arterial calcification, leaving a total of 3265 participants. Of them, 56.5% were women and 43.5% were men. The most prevalent CVRF was hypercholesterolaemia with a prevalence of 47%, followed by overweight (45.9%), high blood pressure (44.4%), obesity (36.8%), MS (21.1%), smoking (16.8%), and DM2 (14.2%) [33].

Participants were considered hypertensive if they had a previous diagnosis, were under treatment, or had a high blood pressure >140/90 mmHg. All participants who had a diagnosis of DM, were under treatment, or had a glycosylated blood glucose >7% were considered diabetic. Hypercholesterolaemia was determined when there was a previous diagnosis, the patient was under lipid-lowering treatment, or total cholesterol was above 250 mg/dL. Obesity and overweight were measured according to the BMI classification and abdominal obesity according to the abdominal circumference measurement, considering abdominal obesity if the waist circumference in men was above 102 cm and 88 cm in women. Finally, tobacco use was stated for those participants who reported having smoked one or more cigarettes per day in the last year [2].

Study in Barcelona.

A descriptive cross-sectional study was carried out in an urban health centre located in a suburb of the city of Barcelona. A total of 2248 participants over 15 years of age were obtained, of whom 53.5% were females and 46.5% were males [34].

The age range with the highest participation response was 65–74 years, and with little difference, 25–34 years. Regarding CVRFs, the most prevalent was smoking (35.2%), followed by high blood pressure (33.7%), obesity (32,7%), hypercholesterolaemia (21.9%), DM2 (15.8%), and hypertriglyceridaemia (12.7%). When disaggregating by sex, smoking was the most prevalent CVRF in men, but in women it was high blood pressure. In addition, the remaining CVRFs were more prevalent in men than in women. Regarding women, the most prevalent CVRF was high blood pressure, with a prevalence of 34.5%, followed by smoking (25.7%), hypercholesterolaemia (23.9%), DM2 (14%), and hypertriglyceridaemia (8.9%). In men, the most prevalent CVRF was smoking, with a prevalence of 45.3%, followed by high blood pressure (32%), hypercholesterolaemia (19.4%), DM2 (17.5%), and hypertriglyceridaemia (17.3%) [34].

In the case of smoking, the prevalence increased until peaking in those aged between 35 and 44 years, and from there onwards it began to decrease. In the case of hypercholesterolaemia, hypertriglyceridaemia, and DM2, as the age of the participant increased, the prevalence also did so, except in the age group over 74, where it decreased. In the case of high blood pressure, the prevalence increased with age in all age groups [34].

Tobacco use was defined if smoking was recorded in the medical history at least in the last two years. Hypertension was defined as having blood pressure levels greater than or equal to 140/90 mmHg or if high blood pressure was recorded in the medical history. As for hypercholesterolaemia, it was defined as having been previously diagnosed in at least the previous 6 years or with total blood cholesterol levels of 250 mg/dL or higher. Hypertriglyceridaemia was determined if there was a prior diagnosis at least in the previous 6 years or if total cholesterol levels were equal to or higher than 200 mg/dL. DM was considered if there was a prior diagnosis, random blood glucose levels above 200 mg/dL with typical clinical signs, two fasting blood glucose measurements above 126 mg/dL, or with an oral glucose overload with measurement at two hours and value above 200 mg/dL. Finally, obesity was determined based on the BMI classification [34].

Registre GIroni del COR study (REGICOR, Girona).

Cross-sectional population-based cohort study. A total of 3724 participants aged between 35 and 74 years living in the province of Girona were included with complete follow-up, with a participation of 51.9% females and 48.1% males. The prevalence of high blood pressure was presented in this study, with this CVRF being the most prevalent (46.5%) and higher in men than in women (51.7% in men and 45.4% in women). Hypercholesterolaemia had a total prevalence of 35.9% and, in this case, it was higher in the female sex, with a prevalence of 39.8% compared to 36.5% in men. Smoking had a total prevalence of 21.9% and was more prevalent in men (31.2%) than in women (14%). Finally, DM2 had a total prevalence of 14.4%, with a higher prevalence in men (17.2%) than in women (12.9%) [35].

The same definitions used for CVRF in the previous study carried out in Barcelona were applied [35].

HERMEX study (Extremadura) [36,37,38,39,40].

The HERMEX study estimated the prevalence, detection, treatment, and degree of control for classic CVRFs in the population of Extremadura, based on the results from a health area [36].

The total number of participants in the study was 2833 aged 25–79 years, of whom 46.5% were males and 53.5% were females. In both women and men, the most prevalent age range of participation was 35–44 years, and rural origin had a prevalence of 52.2% in men and of 49.9% in women [37].

The prevalence of CVRFs was higher in the male sex, with a clear difference across sexes regarding the prevalence of smoking and alcohol abuse. By sex, the most prevalent CVRF in the male sex was alcohol abuse (63.2%), followed by overweight (46.3%), smoking (40.5%), high blood pressure (39.5%), hypercholesterolaemia (37.9%), MS (36.7%), obesity (36.5%), and DM2 (13.7%). In the female sex, the most prevalent CVRF was hypercholesterolaemia (35%), followed by high blood pressure (33%), overweight (32.5%), MS (30.9%), obesity (30.7%), smoking (26.6%), alcohol abuse (12%), and DM2 (12%). The most prevalent CVRF in the population of Extremadura was overweight, with a prevalence of 38.8%, followed by hypercholesterolaemia (36.2%), alcohol abuse (36.1%), high blood pressure (35.8%), MS (33.6%), smoking and obesity with equal prevalence (33.2%), and DM (12.7%) [37,38,39,40].

Overweight and obesity were determined according to the BMI classification; abdominal obesity was determined by waist circumference, with values above 102 cm in men and 88 cm in women being considered pathological. DM, high blood pressure, and hypercholesterolaemia were determined, firstly, if previously diagnosed, and secondly, if glucose ≥ 126 mg/dL, blood pressure ≥ 140/90 mmHg, and total cholesterol ≥ 240 mg/dL. Tobacco use was defined as regular smoking or having quit less than one year ago [37,39]. A diagnosis of MS required meeting three of the following criteria: fasting blood glucose ≥100 mg/dL or under anti-diabetic treatment; high blood pressure, with blood pressure values greater than or equal to 130/85 mmHg, HDL-C lower than 50 mg/dL in women and lower than 40 mg/dL in men; abdominal circumference greater than or equal to 102 cm in men and 88 cm in women; or triglyceride levels ≥150 mg/dL [40]. Alcohol abuse was based on the reported amount of alcohol consumed per week [38].

Prevalence of Cardiovascular Risk Factors in the Balearic Islands (CORSAIB Study, Balearic Islands).

The CORSAIB study assessed the prevalence of CVRFs in the Balearic Islands. A total of 1685 participants aged 35–74 years were included, of whom 52% were females and 46% were males. In both sexes, the most prevalent age range of participation corresponded to the 35–44 years group [41].

CVRFs were expressed by age range and sex. It can be observed that smoking was more prevalent in both sexes in the 35–44 age group, with its prevalence always being higher in men (36.9%) than in women (18.7%); this prevalence decreased with age. As far as high blood pressure is concerned, the most prevalent age group was over 65 years. There was an overall higher prevalence of the disease in men, with the exception of the over-65 age group, where the prevalence was higher in women. The overall prevalence of high blood pressure in men was 52.3% and in women, 43.4%. Hypercholesterolaemia was more prevalent in men aged 45–54 and in women aged 55–64. This prevalence also varied across the two sexes, being slightly higher in men, with a difference of 0.3%, and being more prevalent in men aged 35–54 years and in women aged 55 years and older; the overall prevalence was 24.4% in men and 24.1% in women. The prevalence of diabetes also increased with age, and was more prevalent in both sexes in the population group over 65 and higher in men (15%) than in women (8.4%). Obesity was more prevalent in the female sex, being more pronounced in the population group over 65 years, with the exception that it was higher in the group of men aged 45–54 years. The prevalence of overweight was 48.3% in men and 33.4% in women. Moreover, 43.1% of the men and 45.6% of the women interviewed were sedentary [41].

The total prevalence of CVRFs was 47.8% for high blood pressure, the most prevalent CVRF, followed by sedentary lifestyle (44.4%), overweight (40.1%), smoking and obesity (27%), hypercholesterolaemia (24.2%), and DM2 (11.7%) [41].

Participants were considered hypertensive if they were under antihypertensive treatment or if their blood pressure was higher than 140/90 mmHg. Diabetic patients were those under hypo-glycaemic treatment or who had had an analytical determination with a fasting glucose value higher than 126 mg/dL. Hypercholesterolaemia was determined with total cholesterol values above 250 mg/dL. As for smoking, participants were considered smokers if they reported having smoked in the last six months. As for obesity, it was measured according to the BMI classification. Finally, participants who reported not performing regular physical activity in their free time were considered sedentary [41].

Prevalence of diabetes mellitus and cardiovascular risk factors in the adult population of the autonomous region of Madrid (Spain): the PREDIMERC study.

A cross-sectional population-based study was carried out in 2007, for which a random sample representative of the adult population of the autonomous region of Madrid was selected. The sample included a total of 2268 participants aged between 30 and 74 years. A total of 52% were females and 48% were males [42,43].

The prevalence of CVRFs in the PREDIMERC study varied by sex. Sedentary lifestyle was more prevalent among women (87.5%). However, the remaining CVRFs were more prevalent among men. In women, the CVRFs in order of prevalence were sedentary lifestyle, followed by smoking (25.7%), high blood pressure (23.9%), hypercholesterolaemia (22.3%), obesity (19.8%), overweight (18.1%), diabetes (6%), and hypertriglyceridaemia (5%). In men, sedentary lifestyle was also the most prevalent CVRF (83.4%), followed by high blood pressure (35.1%), smoking (31.4%), overweight (27.9%), hypercholesterolaemia (24.2%), obesity (23.6%), hypertriglyceridaemia (12.7%), and diabetes (10.2%). The most prevalent CVRF was sedentary lifestyle, with a prevalence of 85.5% in the total studied population, followed by high blood pressure (29.3%), smoking (28.4%), hypercholesterolaemia (23.2%), overweight (22.8%), obesity (21.7%), hypertriglyceridaemia (8.8%), and DM2 (8.1%) [42,43].

Patients were considered hypertensive if they were diagnosed with high blood pressure or had self-referred high blood pressure, were under antihypertensive treatment, or if their blood pressure exceeded 140/90 mmHg. Diabetics were those diagnosed, under treatment, or with fasting glucose levels above 126 mg/dL. Hypercholesterolaemia was determined when the patient was taking lipid-lowering drugs or had total cholesterol values of 240 mg/dL or higher [43]. For the diagnosis of overweight and obesity, the BMI classification was used as a reference, based on a diagnosis of overweight grade II when the BMI was greater than 27; for the diagnosis of abdominal obesity, the abdominal circumference measurement was taken as a reference, with values greater than 88 cm for women and 102 cm for men considered pathological. Tobacco use was defined by regular smoking. As for sedentary lifestyle, it was described as not engaging in moderate activity or as being seated for most of the time [42,43].

Diabetes, Nutrición y Obesidad (Programa DINO, Murcia) (Diabetes, Nutrition, and Obesity study (DINO Programme, Murcia).

The DINO study was based on a sample population from the region of Murcia of 1556 participants over the age of 20, of whom 53.8% were females and 46.2% were males. The most prevalent CVRF was sedentary lifestyle (66.7%), followed by overweight (41.6%), smoking (34.3%), obesity (22.6%), hyperlipidaemia (21.6%), high blood pressure (20%), and DM2 (7.8%). When disaggregating by sex, both hyperlipidaemia and high blood pressure were more prevalent among women, the difference being greater in the case of hyperlipidaemia, with a respective prevalence of 24.7% and 19.7% compared to the prevalence in men (21.6% and 19.3%, respectively). Finally, DM2 was more prevalent among men (8.6% compared to a prevalence of 7% in women) [44].

Subjects were considered diabetic if they had fasting blood glucose levels greater than 126 mg/dL or were under treatment. Participants were considered hypertensive if their blood pressure was above 140/90 mmHg or if they were under treatment. As for hyperlipidaemia, it was determined by total cholesterol and triglyceride levels above 200 mg/dL or if being treated with lipid-lowering drugs. Overweight and obesity were measured according to the BMI classification and sedentary lifestyle was measured based on the reported hours of physical activity per week. Finally, tobacco use was measured as reported by the smoker [44].

Proyecto Riesgo Vascular de Navarra (Navarra Vascular Risk Project) study (RIVANA, Navarra) [45,46,47].

The RIVANA study determined the prevalence of CVRFs and MS, their association with subclinical atherosclerotic lesions, and their impact on cardiovascular and cerebrovascular disease at 10 years. The results of the first phase of the study were published in 2007. After the sample selection, a total of 4168 study participants aged between 35 and 84 years were obtained. A total of 45.4% were males and 54.6% were females [45].

With regard to prevalence rates of the assessed CVRFs, it is observed that these are higher in men than in women, with a much greater difference in the case of high blood pressure, with a prevalence of 50.9% in men and 39.4% in women. This was also the most prevalent CVRF in both sexes, followed by hypercholesterolaemia, which was higher in women (31.6%) than in men (30.9%) and had a total prevalence of 41.3%; MS, which occurred more in men (22.1%) than in women (17.2%) and with a total prevalence of 19.4%; and hypertriglyceridaemia and DM2, which showed equal total prevalence rates of 8.5%. However, when disaggregating by sex, the prevalence of both CVRFs (hypertriglyceridaemia and DM2) was higher among men, with 12.7% for hypertriglyceridaemia and 11.1% for DM2 in the case of men and 6.4% for DM2 and 5% for hypertriglyceridaemia in the case of women. The only CVRF where the prevalence was slightly higher in women than in men was hypercholesterolaemia [45].

In 2009, the prevalence of CVRFs was also reported, showing that the total prevalence of overweight and obesity was 65.5%, reaching 77.3% in men and 55.7% in women [46].

A study published in 2020 followed up on the RIVANA study cohort and, in this case, of the 4168 participants who were followed up on to 2017, the sample was reduced to 3976 participants after excluding those who had a major CVD (AMI or stroke). Of these 3976 participants, 45% were males and 55% were females [47].

Regarding the prevalence of CVRFs, the most prevalent was regular alcohol consumption (46%), followed by smoking (39%), hypercholesterolaemia (38%), MS, high blood pressure (25%), hypertriglyceridaemia (19%), and diabetes (9%) [47].

Hypertension was determined for individuals with a previous diagnosis, under treatment, or with blood pressure values of 140/90 mmHg or higher. Hypercholesterolaemia was considered in participants with a previous diagnosis, under treatment, or with total cholesterol values greater than or equal to 250 mg/dL. Participants with a diagnosis of diabetes, under treatment, or with altered basal blood glucose levels between 100 and 125 mg/L or higher than 126 mg/dL were considered diabetic. The measurement of overweight and obesity was based on the BMI classification, and abdominal obesity was measured through waist circumference, based on pathological figures higher than 102 cm in men and 88 cm in women [46]. The diagnosis of MS was conducted on the basis of the American Heart Association definition, where the individual had to meet at least three of the following criteria: abdominal circumference above 94 cm in men and 80 cm in women; blood pressure greater than or equal to 130/85 mmHg or under treatment; basal glucose above 100 mg/dL or under treatment; triglycerides above 150 mg/dL or under treatment; and HDL-C below 50 mg/dL in men or 40 mg/dL in women [45,47].

### 3.3. Risk of Bias

Each study was selected through critical reading with the CASPe tool for cohort studies [11], the Berra et al. tool for cross-sectional studies [12], and the synthesis of the evidence with degrees of recommendation in order to minimise potential biases [13]. 

The differences found in the age ranges studied could represent a variation in the prevalence of CVRFs and, therefore, a bias in the studies, given that many factors tend to increase with age. In all cases, there was a higher participation of female subjects; this difference was more pronounced in the Di@bet.es study [21,22,23]. This fact may have caused a selection bias and over-representation of females when extrapolating to the general population. Also, the studied risk factors vary across the different publications.

Despite this, the studies were considered for inclusion as it can be seen that the results obtained were along the same lines in all studies.

### 3.4. Synthesis of the Results

The results of the critical reading, the level of evidence, and the level of recommendation for each of the studies are presented in Table 2.

## 4. Discussion

**Figure 3:**
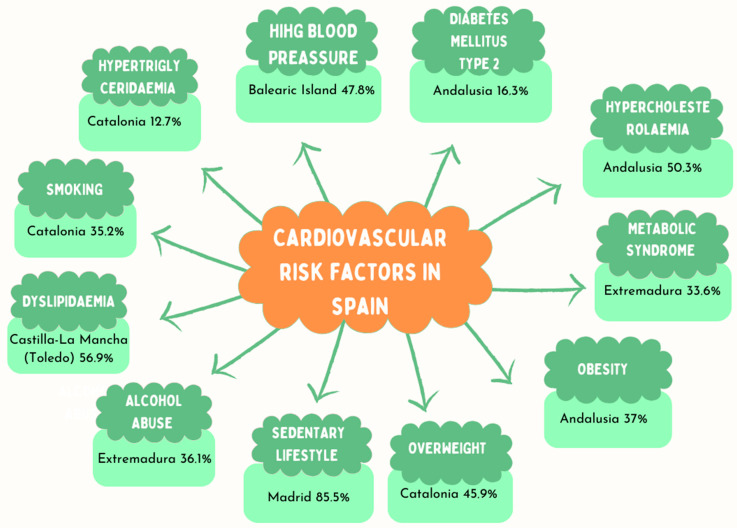
Most prevalent CVRFs in each autonomous region.

Table 4 shows a comparison at the national level of all included studies. Table 5 shows the prevalence of CVRFs by autonomous region. And Figure 3 shows the highest prevalence of every single risk factor in each region considered. 

There are clear population differences in the prevalence of CVRFs by autonomous region. Specific studies are only available for 10 out of 17 existing autonomous regions. Given that there are differences between the values at national level and the regional studies, and across the regional studies, the map of prevalence of CVRFs needs to be completed.

There are also differences regarding the age range of participants in each study. In the RECCyL [28,29,30] and the Barcelona [33] studies, the age for participation was over 15 years; over 18 in the RICARTO [3] and Di@bet.es [21,22,23] studies; over 20 in the DINO [43] study; and over 49 in the ARTPER [31,32] study. The CDC study in the Canary Islands included a population aged between 18 and 75 years [24,25,26,27]. The age range used in the CORSAIB [40], REGICOR [34], TALAVERA [31], and DARIOS [17,18,19,20] studies was 35 to 74 years, widening from 74 to 84 in the RIVANA [44,45,46] study, from 35 to 25 in the HERMEX [35,36,37,38,39] study, and from 35 to 30 in PREDIMERC [41,42].

High blood pressure was the only CVRF present in 100% of the studies, and in the case of DM2, only the TALAVERA [31] study did not analyse its prevalence. Smoking was not assessed in RIVANA [44,45,46] or DARIOS [17,18,19,20], and hypercholesterolaemia in RECCyL [28,29,30], RICARTO [3], Di@bet.es [21,22,23] at national level, DINO [43], or DARIOS [17,18,19,20] in the 2011 publication. Obesity was not assessed in the RIVANA [44,45,46], REGICOR [34], Barcelona [33], or DARIOS 2011 studies [17,18,19,20], and no data on MS were presented in CORSAIB [40], Di@bet.es Andalusia [21,22,23], Barcelona [33], REGICOR [34], or TALAVERA [31]. Overweight data were not included in RECCyL [28,29,30] or Di@bet.es Andalusia [21,22,23]; sedentary lifestyle was not included in RECCyL [28,29,30] or HERMEX [35,36,37,38,39]; alcohol abuse was only studied in CDC [24,25,26,27], RICARTO [3], and HERMEX [35,36,37,38,39]; dyslipidaemia was studied in RICARTO [3], RECCyL [28,29,30], Di@bet.es [21,22,23] at the national level, DINO [43], and DARIOS in the study published in 2011 [17,18,19,20]; and hypertriglyceridaemia was included in the RIVANA [44,45,46], Barcelona [33], PREDIMERC [41,42], and ARTPER [31,32] studies. It should be noted that in the RIVANA study [44,45,46], although isolated prevalence rates of obesity and overweight were not presented, the total prevalence of the overweight and obese population included in the study was indeed reported, yet as a whole.

When looking at the prevalence of CVRFs by sex, there are clear differences. In general, they were more prevalent in men than in women. The most prevalent CVRFs in the female sex were obesity and sedentary lifestyle, although in some autonomous regions there was also a higher prevalence of other factors, such as MS in the case of the Canary Islands [24,25,26,27], high blood pressure in the case of Navarra [44,45,46], high blood pressure and high cholesterol in Toledo [3] and Murcia [43], high blood pressure in Barcelona [33], and high cholesterol in Girona [34]. As for Extremadura [35,36,37,38,39], all the CVRFs included in the study were more prevalent among the male sex. These results may have been affected by the over-representation of women in the study samples.

With regard to the total prevalence of CVRFs, high blood pressure was among the three most prevalent CVRFs in all the studies, with the exception of the HERMEX [35,36,37,38,39] and RICARTO [3] studies, where it appeared fourth, and the DINO [43] study. In the case of CORSAIB [40], RECCyL [28,29,30], REGICOR [34], and TALAVERA [31], it was the most prevalent CVRF of all those studied. In the RIVANA [44,45,46], Barcelona [33], PREDIMERC [41,42], ARTPER [31,32], and DARIOS studies [17,18,19,20], high blood pressure was the second most prevalent factor. In the CDC [24,25,26,27] and Di@bet.es [21,22,23] studies, it was in third place. The next most prevalent CVRF in the top three and in greatest number was overweight, being the most prevalent CVRF in both the DARIOS [17,18,19,20] and HERMEX [35,36,37,38,39] studies. In the case of CDC [24,25,26,27], Di@bet.es [21,22,23], and DINO [43], it was the second most prevalent factor, and in the case of ARTPER [31,32], RICARTO [3], and CORSAIB [40], the third. The next CVRF that was present in the largest number of studies and which also was among the top three was sedentary lifestyle, which was the most prevalent factor in the Canary Islands [24,25,26,27] and Andalusia [21] studies, with this prevalence also being extended to the Di@bet.es study [21,22,23] at the national level, Madrid [41,42], and Murcia [43], and also occupying the second place in the Balearic Islands [40]. Smoking and hypercholesterolaemia were found to be among the top three CVRFs in six of the analysed studies, with smoking being the first one in Barcelona [33] and the third in REGICOR [34], TALAVERA [31], PREDIMERC [41,42], DINO [43], and DARIOS [17,18,19,20]. Hypercholesterolaemia appeared first in the ARTPER [31,32] study, second in Di@bet.es Andalusia [21,22,23], HERMEX [35,36,37,38,39], and REGICOR [34], and third in RIVANA [44,45,46] and Barcelona [33]. Dyslipidaemia was among the top three risk factors in three of the studies, being the most prevalent CVRF in the RICARTO [3] and DARIOS [17,18,19,20] studies and the second most prevalent in the RECCyL study [28,29,30]. Obesity occupied second place in terms of prevalence in the TALAVERA [31] study and third place in the RECCyL study [28,29,30], and alcohol abuse was the third most prevalent factor in Extremadura [35,36,37,38,39]. In the case of Navarra [44,45,46], as prevalence rates of overweight and obesity were not disaggregated, they were studied jointly, both CVRFs being the most prevalent ones in this case. 

By autonomous regions, the region with the highest prevalence of high blood pressure was the Balearic Islands [40]. For DM, obesity, and hypercholesterolaemia, the highest prevalence was found in Andalusia [21]. The highest prevalence of MS was recorded in Extremadura [35,36,37,38,39]. Overweight was most prevalent in Murcia [43]; sedentary lifestyle, in Madrid [41,42]; smoking and hypertriglyceridaemia were more prevalent in Barcelona [33]; and alcohol abuse in Extremadura [35,36,37,38,39]. Finally, dyslipidaemia had a higher prevalence in Toledo [3].

### Limitations of the Study

In the study by Cabrera de Leon, 2008 [24], a total of 6729 participants were reported in the sample recruitment phase, but in the sample presentation table, the total number of participants presented was 6718. Subsequent studies were based on the cohort of 6729 participants [21].

In the case of the Di@bet.es study, according to Rojo-Martinez et al. [22] and Cuesta et al., [23] the results could not yet be generalised as the initial study population had not been fully reassessed. Furthermore, the significant difference between the overall prevalence of low physical activity in Andalusia as opposed to the rest of Spain is noteworthy [22,23].

In the study carried out in Navarra, prevalence rates of overweight and obesity were not presented in an isolated manner, but combined [45]. In addition, slight differences were observed in the prevalence reported in the studies by Viñes et al. [44] and Amezqueta et al. [45] despite relying on the same sample.

In the TALAVERA study, prevalence rates of DM2 and overweight were not presented [31].

In the case of the HERMEX study [35,36,37,38,39], discrepancies were found in terms of prevalence rates for obesity, with a slight difference in two studies despite having analysed the same sample (2833 subjects) [34,35].

The RICARTO study reported the results of the first 1500 cases [3]. Nevertheless, a comparison between the results obtained and the final results of the studies presented above shows that the preliminary results were in line with those already published.

A noteworthy limitation is that only articles in Spanish or English were included in this review. The reason for doing so was that the objective of the search was restricted to results obtained about the Spanish population, so authors were therefore expected to have publish either in Spanish or in English. However, it is assumed that this may have implied a limitation to the search process.

Another limitation identified is the variability in the definition, measurement instruments, and methods of analysis of CVRFs. This, in turn, accounts for the variability observed in prevalence rates. In part, this may account for the differing values found across autonomous regions.

## 5. Conclusions

It can be concluded that the prevalence of CVRFs in Spain varies according to the autonomous region, the individual’s sex, and the age range. In addition, some factors increase in prevalence as they are associated with other CVRFs.

The main recommendation to be drawn from the present study is, from a methodological point of view, to unify the criteria for both the definition and the analysis of the different CVRFs. The absence of prevalence values related to cardiovascular factors according to each Spanish region has motivated this study, as well as further research to be carried out at the national level in order to know current prevalence rates.

## Figures and Tables

**Table 1:** Research question with PICO format.

P	I	C	O
Adult population over 18 years of age	Estimation of CVRF prevalence	Cardiovascular risk factors	Prevalence of CVRFs

**Table 2:** Search strategy.

Search Engine	Date	Mesh/Decs	Total Articles	Selected Articles (Title/Abstract)	Selected Articles (Critical Reading)
Pubmed	8 August 2023	[Heart Disease Risk Factors AND Prevalence AND Spain] [Factores de Riesgo de Enfermedad Cardiaca AND Prevalencia AND España]	6767	80	37
Pubmed	8 August 2023	[Heart Disease Risk Factors AND Prevalence AND Spain] [Factores de Riesgo de Enfermedad Cardiaca AND Prevalencia AND España]	2630	51	25
VHL *	8 August 2023	[Heart Disease Risk Factors AND Prevalence AND Spain] [Factores de Riesgo de Enfermedad Cardiaca AND Prevalencia AND España]	2702	71	28
VHL	8 August 2023	[Heart Disease Risk Factors AND Prevalence AND Spain] [Factores de Riesgo de Enfermedad Cardiaca AND Prevalencia AND España]	1605	43	36

* Virtual Health Library; limits: publications in Spanish/English.

**Table 3:** Critical reading, levels of evidence, and degrees of recommendation.

Reference	Type of Study	Critical Reading	Synthesis of the Evidence **
Cuesta et al., 2020 [23]	Prospective cohort study	10/10 * p < 0.05	LE: 2++ DR: B
Rojo-Martínez et al., 2020 [22]	Cross-sectional study	High quality ** p < 0.001–0.3	LE: 2++ DR: B
Valdés et al., 2014 [21]	Cross-sectional study	High quality ** p < 0.001–0.01	LE: 2++ DR: B
Rodríguez-Roca et al., 2018 [3]	Epidemiological, observational, and analytical study	High quality ** p < 0.001	LE: 2++ DR: B
Fernández-Bergés et al., 2011 [40]	Cross-sectional, descriptive	High quality ** p < 0.001	LE: 2++ DR: B
Félix-Redondo et al., 2011 [37]	Cross-sectional, descriptive, observational	High quality ** p < 0.001–0.217	LE: 2++ DR: B
Félix-Redondo et al., 2012 [39]	Cross-sectional, descriptive	High quality ** p < 0.001–0.913	LE: 2++ DR: B
Félix-Redondo et al., 2012 [38]	Cross-sectional, descriptive	High quality ** p < 0.678	LE: 2++ DR: B
Rigo-Carratalá et al., 2005 [41]	Cross-sectional, descriptive	High quality **	LE: 2++ DR: B
Baena-Díez et al., 2005 [35]	Cross-sectional, descriptive	High quality ** p < 0.05	LE: 2++ DR: B
Baena-Díez et al., 2011 [17]	Pooled analysis with individualised data from 11 studies (cross-sectional study)	High quality ** p < 0.01–0.024	LE: 2++ DR: B
Baena-Díez et al., 2014 [18]	Pooled, cross-sectional study	High quality **	LE: 2++ DR: B
Fernández-Bergés et al., 2012 [19]	Pooled analysis with individualised data from 11 studies (cross-sectional study)	High quality ** p < 0.001	LE: 2++ DR: B
Félix-Redondo et al., 2013 [20]	Pooled analysis with individualised data from 11 studies (cross-sectional study)	High quality ** p < 0.001	LE: 2++ DR: B
Cabrera de León et al., 2008 [24]	Prospective study	10/10 * p < 0.001–0.005	LE: 2++ *** DR: B ***
Cabrera de León et al., 2009 [25]	Cross-sectional study	High quality ** p < 0.01–0.005	LE: 2++ DR: B
Cuevas-Fernández et al., 2020 [26]	Follow-up study	9/10 * p < 0.011–0.001	LE: 2++ DR: B
González-Romero et al., 2016 [27]	Follow-up study	9/10 * p < 0.001–0.065	LE: 2++ DR: B
Gil-Montalbán et al., 2010 [42]	Cross-sectional study	High quality ** p < 0.00	LE: 2++ DR: B
Ortiz-Marrón et al., 2011 [43]	Cross-sectional study	High quality ** p < 0.01–0.05	LE: 2++ DR: B
Segura-Fragoso et al., 1999 [31]	Cross-sectional study	High quality ** p < 0.05	LE: 2++ DR: B
Alzamora et al., 2010 [32]	Cross-sectional study	High quality ** p < 0.001–0.376	LE: 2++ DR: B
Baena-Díez et al., 2005 [34]	Cross-sectional study	High quality ** p < 0.05	LE: 2++ DR: B
Vega-Alonso et al., 2007 [28]	Cross-sectional study	High quality **	LE: 2++ DR: B
Vega-Alonso et al., 2008 [29]	Cross-sectional study	High quality ** p < 0.01–0.98	LE: 2++ DR: B
Escribano-García et al., 2011 [30]	Cross-sectional study	High quality ** p < 0.01–0.268	LE: 2++ DR: B
Huerta et al., 2009 [44]	Cross-sectional study	High quality ** p < 0.01–0.997	LE: 2++ DR: B
Viñes et al., 2007 [45]	Cross-sectional study	High quality ** p < 0.0016–0.9257	LE: 2++ DR: B
Guembe et al., 2020 [47]	Prospective study	10/10 *p* < 0.313–0.853	LE: 2++ DR: B
Amézqueta et al., 2009 [46]	Cross-sectional study	High quality **	LE: 2++ DR: B
Alzamora et al., 2012 [33]	Cross-sectional study	High quality ** p < 0.01–0.567	LE: 2++ DR: B
Forés et al., 2017 [2]	Prospective cohort study	10/10 * p < 0.000–0.565	LE: 2++ DR: B
Marrugat et al., 2011 [36]	Follow-up study	10/10 * p < 0.001–0.883	LE: 2++ DR: B

* CASPe for cohort studies; ** Berra et al. for cross-sectional studies; *** according to SIGN; level of evidence (LE); degree of recommendation (DR); reviewers: SP, SG, DC.

**Table 4:** Comparison of studies at national level.

	DARIOS 2011 (National)	DARIOS 2014 (National)	Di@bet.es Spain 2020 (National)	Di@bet.es Andalusia	CDC (Canary Islands)	RECCyL (Castilla-Leon)	TALAVERA (Castilla-La Mancha)	RICARTO (Toledo)	ARTPER (Catalonia)	Barcelona (Catalonia)	REGICOR (Catalonia)	HERMEX (Extremadura)	CORSAIB (Balearic Islands)	PREDIMERC (Madrid)	DINO (Murcia) (Murcia)	RIVANA (Navarra)
Total population	27,903	17,291	5072 (2408 **)	1517	6718	4012	1330	3125 (1500 **)	3786 (3265 **)	2248	3724	2833	1685	2268	1556	4168
Males (%)	46	47.1	39.7	35.1	43	48.1	47.3	44.4	46.5	46.5	48.1	46.5	48	48	46.2	45.4
Females (%)	54	52.9	60.3	64.9	57	51.9	52.7	55.6	53.5	53.5	51.9	53.5	52	52	53.8	54.6
Age	35–74	35–74	>18	>18	18–75	>15	25–74	>18	>49	>15	35–74	25–79	35–74	30–74	>20	35–84
High blood pressure (%)	29.3	35.2	38.8	43.9	37.3	38.7	41.5	33	44.4	33.7	46.5	35.8	47.8	29.3	20	44.6
DM2 (%) *	13.6	13.2	6.5	16.3	10.6	12.3	--- ***	8.6	14.2	15.8	14.4	12.7	11.7	8.1	7.8	8.5
Hypercholesterolaemia (%)	---	37	---	50.3	31.4	---	15.7	---	47	21.9	35.9	36.2	24.2	23.2	---	41.3
Metabolic syndrome (%)	---	---	39	---	24	20.5	---	20.1	21.1	---	---	33.6	---	---	---	19.4
Obesity (%)	---	29.6	26.2	37	28.1	27.1	29.9	25.3	36.8	32,8	---	33.2	27	21.7	22.6	65.5 ****
Overweight (%)	---	43	42.2	---	38.2	---	---	39.1	45.9	---	---	38.8	40.1	22.8	41.6	---
Sedentary lifestyle (%)	---	---	42.3	58.9	64	---	---	39.4	---	---	---	---	44.4	85.5	66.7	---
Smoking (%)	---	22.9	24.4	29	25.3	20.6	25.9	24.2	16.8	35.2	21.9	33.2	27	28.4	34.3	---
Alcohol abuse (%)	---	---	---	---	6.7	---	---	2.3	---	---	---	36.1	---	---	---	---
Dyslipidaemia (%)	33.	---	26.6	---	---	34	---	56.9	---	---	---	---	---	---	21.6	---
Hypertriglyceridaemia (%)	---	---	---	---	---	---	---	---	---	12.7	---	---	---	8.8	---	8.5

* Diabetes mellitus type 2; ** study population; *** no data to show; not included in the study; **** reports percentage of overweight and obesity as a whole.

**Table 5:** Prevalence of CVRFs by autonomous region.

	High Blood Pressure (%)	DM2 * (%)	Hypercholesterolaemia ** (%)	Metabolic Syndrome (%)	Obesity (%)	OverWeight (%)	Sedentary Lifestyle (%)	Smoking (%)	Alcohol Abuse (%)	Dyslipidaemia (%)	Hypertriglyceridaemia (%)
National level **	29.3–38.8	6.5–13.6	37	39	26.2–29.6	42.2–43	42.3	22.9–24.4	--- ***	26.6–33	---
Andalusia	43.9	16.3	50.3	---	37	---	58.9	29	---	---	---
Canary Islands	37.3	10.6	31.4	24	28.1	38.2	64	25.3	6.7	---	---
Castilla-Leon	38.7	12.3	---	20.5	27.1	---	---	20.6	---	34	---
Castilla-La Macha ****	33–41.5	8.6	15.7	20.1	25.3–29.9	39.1	39.4	24.2–25.9	2.3	56.9	---
Catalonia	44.4–46.5	14.2 15.8	21.9–47	21.1	32.8–36.8	45.9	---	16.8–35.2	---	---	12.7
Extremadura	35.8	12.7	36.2	33.6	33.2	38.8	---	33.2	36.1	---	---
Balearic Islands	47.8	11.7	24.2	---	27	40.1	44.4	27	---	---	---
Madrid	29.3	8.1	23.2	---	21.7	22.8	85.5	28.4	---	---	8.8
Murcia	20	7.8	---	---	22.6	41.6	66.7	34.3	---	21.6	---
Navarra	44.6	8.5	41.3	19.4	65.5 *****	---	---	---	---	---	8.5

* DM2: diabetes mellitus type 2; ** data from DARIOS and Di@bet.es studies; *** no data to show; not included in the study; **** data from TALAVERA and RICARTO studies; ***** reports percentage of overweight and obesity as a whole.

## Data Availability

All data have been included within this article.
